# Narrowing down the single homoeologous *FaPFRU* locus controlling flowering in cultivated octoploid strawberry using a selective mapping strategy

**DOI:** 10.1111/pbi.12574

**Published:** 2016-06-09

**Authors:** Justine Perrotte, Amèlia Gaston, Aline Potier, Aurélie Petit, Christophe Rothan, Béatrice Denoyes

**Affiliations:** ^1^ INRAUMR 1332 BFPVillenave d'OrnonFrance; ^2^ Université de BordeauxUMR 1332 BFPVillenave d'OrnonFrance; ^3^ Ciref Création Variétale Fraises Fruits RougesDouvilleFrance; ^4^ Present address: CNRS, UMR 5200Laboratoire de Biogenèse MembranaireUniversité de BordeauxBordeauxFrance

**Keywords:** *Fragaria*, polyploidy, perpetual flowering, bin mapping, marker‐assisted selection

## Abstract

Extending the period of fruit production is a way to substantially increase crop yield in many fruit or ornamental species. In the cultivated octoploid strawberry (*Fragaria × ananassa*), the most consumed small fruit worldwide, fruit production season can be extended by selecting the perpetual flowering (PF) cultivars. This trait is of considerable interest to growers and to the food industry. Four homoeologous loci controlling a single trait can be expected in such a complex octoploid species. However, we recently showed that the PF trait is under the control of the single dominant *FaPFRU* locus (*J. Exp. Bot*., 2013, **64**, 1837), making it potentially amenable to marker‐assisted selection (MAS). Here, we report the successful use of a strategy, based on a selective mapping using a reduced sample of individuals, to identify nine markers in close linkage to the *FaPFRU* allelic variant. Thus, this strategy can be used to fine map the target homoeologous loci in other complex polyploid crop species. Recombinant analysis further enabled us to reduce the locus to a region flanked by two markers, Bx083_206 and Bx215_131, corresponding to a 1.1 Mb region in the diploid *F. vesca* reference genome. This region comprises 234 genes, including 15 flowering associated genes. Among these, the *FLOWERING LOCUS T* (*FT*) is known to be a key activator of flowering. The close association between the PF trait and the *FaPFRU* flanking markers was validated using an additional segregating population and genetic resources. This study lays the foundation for effective and rapid breeding of PF strawberry cultivars by MAS.

## Introduction

The length of the flowering period is a key trait in the development of high‐yielding fruit or ornamental crops, because it determines flower number and, consequently, reproductive success. This trait depends on floral initiation, which is controlled by both environmental and genetic factors. Commonly for polycarpic perennial plants, floral initiation happens once a year (‘seasonal flowering’, SF). However, some perennials have the ability to flower more than once, thereby prolonging a favourable season, through a process called ‘remontancy’ or ‘perpetual flowering’ (PF) (Albani and Coupland, 2010; de Camacaro *et al*., 2002). The promise of enhancing fruit production by increasing the flowering period makes this trait a key objective of numerous breeding programmes.

Perpetual flowering control has recently been described for rose and woodland diploid strawberry (*Fragaria vesca*) where, respectively, transposon insertion and mutation lead to the inactivation of a homologue of the floral repressor *TFL1* (Iwata *et al*., 2012; Koskela *et al*., 2012). For the cultivated octoploid strawberry, the PF trait has been shown to be controlled by the major QTL *FaPFRU* locus, which has been mapped to the female linkage group IVb (LGIVb‐f) (Castro *et al*., 2015; Gaston *et al*., 2013). The allelic variant is dominant and confers the PF behaviour to the plant with a negative effect on the production of stolons, which are specialized branch crowns producing new plants (the so‐called runnering process) (Savini *et al*., 2008). A fine mapping of this region has been impeded by the octoploid status of the cultivated strawberry, and thus, it remains a challenging task.

Polyploidy resulting from whole‐genome duplications has occurred in many flowering plants (Hegarty and Hiscock, 2008; Jiao *et al*., 2011), including major crops, such as wheat, rape, sugarcane and cultivated strawberry. This process has long been recognized as a major force in evolution (Ohno, 1970). Polyploidization is usually followed by a process of diploidization (Bowers *et al*., 2003; Paterson *et al*., 2004), whereby gene redundancy is reduced via gene silencing, sequence elimination or rearrangement, demethylation of retroelements and relaxation of imprinting (see review in Soltis *et al*., 2015). Although our understanding of the consequences of polyploidization has considerably increased in the past decades (Conant *et al*., 2014), high ploidy level has undoubtedly delayed the development of genomic tools for certain crop species. Consequently, few genomes of polyploid species have been sequenced (allotetraploid rape: Chalhoub *et al*., 2014; hexaploid wheat: Mayer *et al*., 2014; allotetraploid cotton: Zhang *et al*., 2015). The virtual ‘reference genome’ composed of 211 588 sequences was the first attempt to provide the sequence of the cultivated octoploïd strawberry (Hirakawa *et al*., 2014). These sequences, which cover 70.3% of the total length of the *F. vesca* genome (v1.1), highlight the high level of macrosynteny within *Fragaria* genomes. This high level of macrosynteny and of colinearity between the *Fragaria* genomes was previously shown through the comparative mapping approaches (Isobe *et al*., 2013; Rousseau‐Gueutin *et al*., 2008; Sargent *et al*., 2009). However, rearrangements and duplications can be found within specific regions (van Dijk *et al*., 2014; Goldberg *et al*., 2010; Sargent *et al*., 2009; Spigler *et al*., 2010). In addition, the cultivated strawberry displays a major disomic behaviour at meiosis (Lerceteau‐Köhler *et al*., 2003; Tennessen *et al*., 2014). The diploid reference genome provides a reliable tool for fine mapping and positional cloning in the cultivated octoploid strawberry (Lerceteau‐Köhler *et al*., 2012; Rousseau‐Gueutin *et al*., 2008; Weebadde *et al*., 2008).

Here, we present a strategy based on selective mapping and further refined with QTL identification and fine mapping that has successfully pinpointed the single *FaPFRU* locus controlling the PF trait in a genetically complex octoploid species of strawberry. Consequently, markers in close linkage to the *FaPFRU* locus have been identified. Moreover, our ability to transfer markers to a new segregating population and to 17 PF accessions indicates a single origin of the PF trait in modern strawberry cultivars. These findings will lead to the efficient and rapid breeding of strawberry PF cultivars by the marker‐assisted selection (MAS). In addition, we have shown that 15 flowering associated genes are present within the refined locus. Among these genes is the *FLOWERING LOCUS T* (*FT*) gene that is known to be a key activator of flowering.

## Results

### The strategy to fine map a specific locus in the genetically complex polyploid species *Fragaria × ananassa*

**Figure 1:**
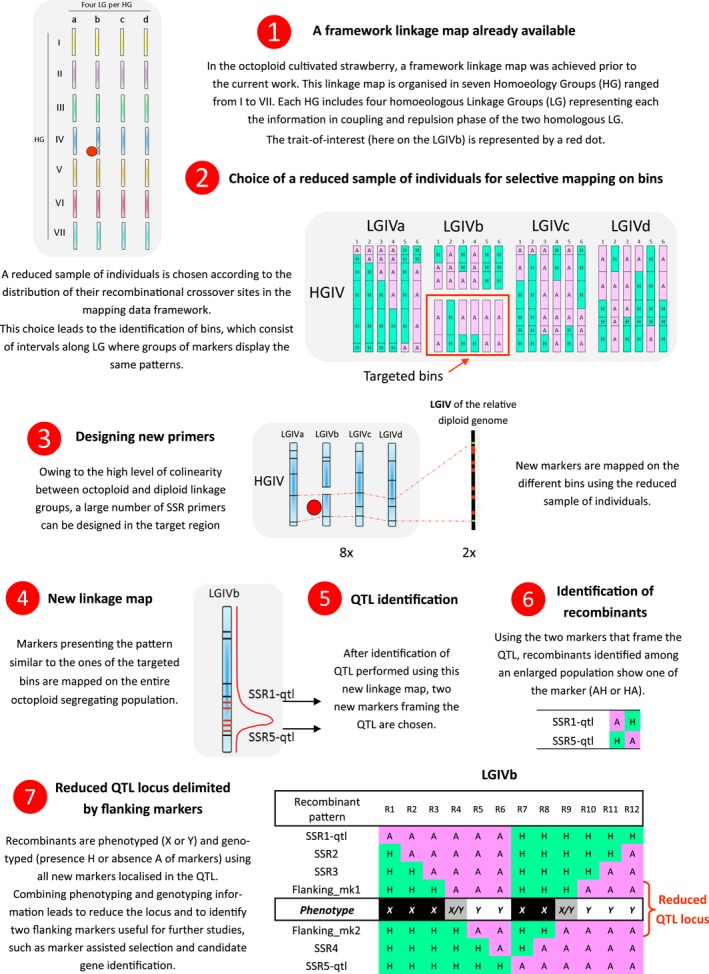
The overall strategy in seven steps based on selective mapping using a reduced sample of individuals and combined with QTL identification and fine mapping.

In the cultivated octoploid strawberry, each locus is represented eight times in the genome that is eight homoeologous alleles can be found. When a major locus shows a 1:1 segregation in test cross, only one out of the eight homoeologues carries the allele of interest. Efforts to fine map a single specific allele at the exclusion of the remaining seven is very challenging. To overcome these limitations, we have developed a strategy that combines several complementary approaches (Figure 1). This strategy uses a previously described strawberry framework map (Lerceteau‐Köhler *et al*., 2012) that facilitates the identification of a reduced number of individuals for selective mapping (Vision *et al*., 2000). New markers are then developed in the genomic region of interest, exploiting the colinearity between the genomes of the octoploid and diploid strawberries (Shulaev *et al*., 2011). The markers are subsequently used to redefine the boundaries for the locus of interest and to identify recombinants in larger populations that segregate for the trait. When applied to a single homoeologous locus, this strategy leads to reduction in and refinement of the locus.

### Reducing the sample size of individuals optimizes the mapping of simple sequence repeat (SSR) markers at the *FaPFRU* locus

**Figure 2:**
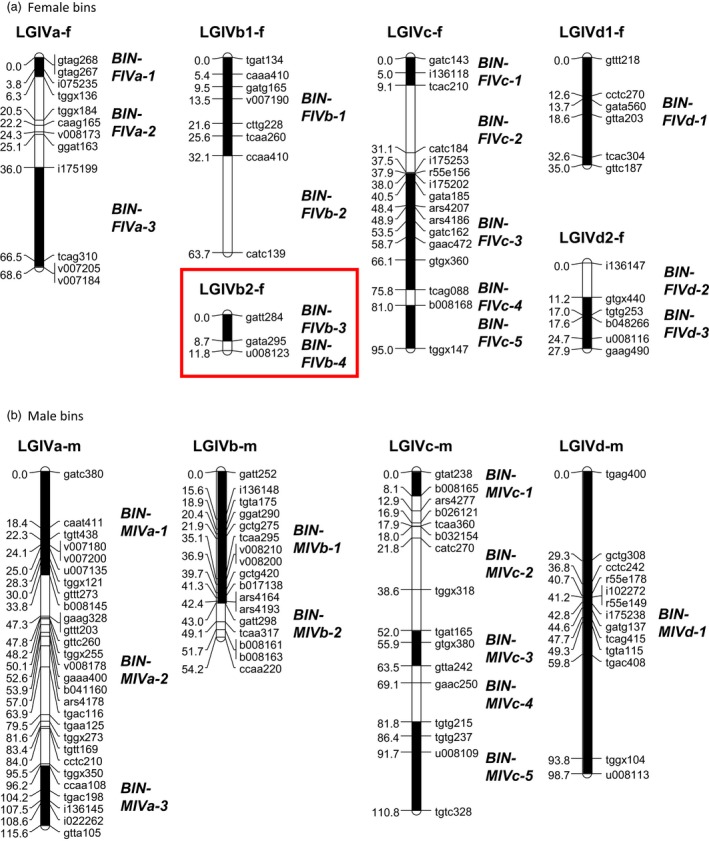
Representation of the bins on (a) the four female and (b) male linkage groups (LG) belonging to the homoeologous group IV. Bins consist of groups of markers separated by recombinational crossover sites. The position of each bin is shown by a black or white rectangle on the LG, and the name of each bin is indicated in italic. The targeted bins overlapping the *FaPFRU* locus are framed in red.

The parents and the 213 progeny comprising the reference segregating population resulting from the cross between ‘Capitola’ and ‘CF1116’ (Table 1) (Lerceteau‐Köhler *et al*., 2003; Rousseau‐Gueutin *et al*., 2008) were the source of data, underlying the mapping framework (Figure 1, Step 1). The reduced number of individuals was chosen according to the distribution of their crossover sites of recombination, also referred to as breakpoints (Vision *et al*., 2000). The breakpoints define bins, which are intervals along a linkage group (LG) lacking a recombinational crossover site for a given set of individuals and that are bounded by breakpoints in at least one individual or by the end of a LG (Vision *et al*., 2000) (Figure 1, Step2). Because the *FaPFRU* locus is located at the bottom of LGIVb‐f (Gaston *et al*., 2013), we optimized the representation of the breakpoints to the bottom of the homoeologous group (HG) IV in the female map HGIV‐f, which includes the four LGs, LGIVa‐f, LGIVb‐f, LGIVc‐f and LGIVd‐f. The final sample of individuals included the parents of the segregating population and six individuals, ‘Capitola’ × ‘CF1116’ 35, 62, 70, 75, 118 and 128. Selection was performed using the MapPop program (Vision *et al*., 2000), which was further refined by visual inspection. The objective was to introduce recombination between the two markers located at the LGIVb‐f ends, gatt284 and gata295, to link the two sub‐linkage groups (Figure 2).

**Table 1:** Populations studied for their recombination in the *FaPFRU* locus. Recombinants, number and percentage, between the flanking markers Bx089_196 and Bx064_216 were identified for each population

	No. of seedlings	No. recombinants			Recombinant (%)
		Bx089_196–Bx064_216		Total recombinants	
		0–1	1–0		
Populations from cross					
‘Capitola’ × ‘CF1116’	372	20	9	29	7.8
‘Capitola’ × ‘Pajaro’	212	5	5	10	4.7
‘Mara des Bois’ × ‘Pajaro’	85	6	2	8	9.4
Total in additive configuration	669	31	16	47	7.0
Populations from selfing					
S_1_ populations					
‘Capitola’‐S_1_	432	18	17	35	8.1
‘Mara des Bois’‐S_1_	210	7	13	20	9.5
F_2_ populations					
[‘Mara des Bois’ × ‘Pajaro’]F_1_‐no. 3	56	1	0	1	1.8
[‘Mara des Bois’ × ‘Pajaro’]F_1_‐no. 45	84	0	1	1	1.2
Total in co‐dominant configuration	782	26	31	57	7.3
Total	1451	57	47	104	7.2

‘Capitola’ × ‘CF1116’ (210 individuals out of the 372) and ‘Mara des Bois’ × ‘Pajaro’ were the two mapping populations.

Within this reduced sample, the four female LGs belonging to HGIV, LGIVa‐f, LGIVb‐f, LGIVc‐f and LGIVd‐f were divided into three, four, five and three bins, respectively, yielding a total of 15 bins. Bin construction resulted in an average length of 11.2 cM per bin (Figure 2 and Table S1). The longest bin identified was BIN‐FIVc‐3 that spanned 35.1 cM. Furthermore, this reduced sample produced a male map divided into 11 bins (Figure 2 and Table S1) that was used to rapidly map a large number of SSR markers.

### Development of SSR primers for the fine mapping of the *FaPFRU* locus

**Figure 3:**
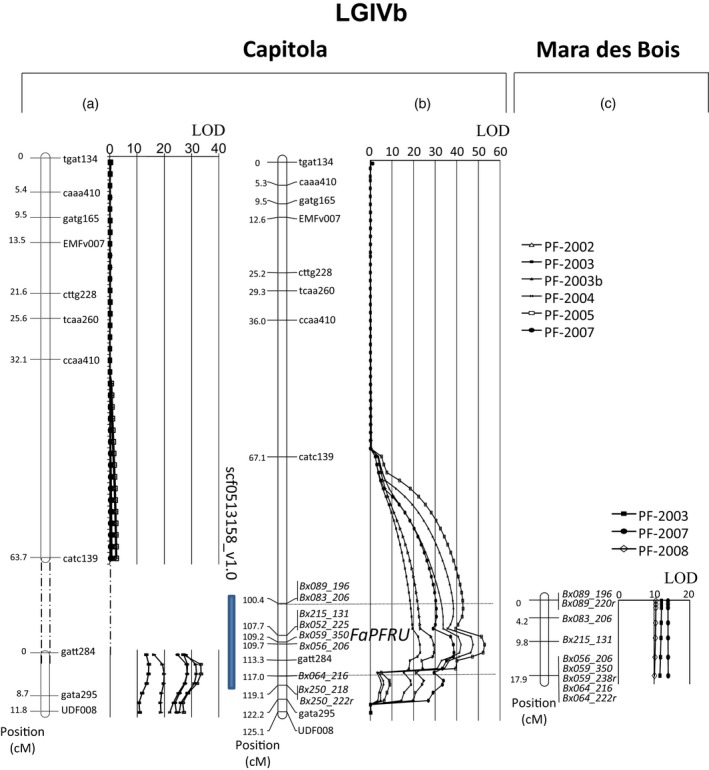
QTLs linked to perpetual flowering (PF) trait identified in ‘Capitola’: (a) in Gaston *et al*. (2013) and (b) in this study and (c) in ‘Mara des Bois’ on the Linkage Group LGIVb. The blue bar represents the position of the scaffold scf0513158_v1.0. Phenotypic data analysed for QTL identification are the number of newly emerged inflorescences from the end of May to the beginning of August, representing the PF trait, for 6 and 3 years, respectively ‘Capitola’ × ‘CF1116’ −(a) and (b)− and ‘Mara des Bois’ × ‘Pajaro’ −(c)− segregating populations. Markers added in this study are in italic.

For developing new markers, we focused on the genomic region of the diploid reference genome upstream of the UDF008 marker (Gaston *et al*., 2013) (Figure 1, Step3). This region is orthologous to the *FaPFRU* locus (Figure 3a) and corresponds to the large scaffold scf0513158_v1.0 (4 198 144 bp) (Shulaev *et al*., 2011; GDR, http://www.rosaceae.org). Based on this scaffold sequence, a total of 275 novel, non‐redundant SSR primer pairs were developed leading to an average spacing of one SSR per every 14 000 Kb of sequence. In addition, 16 SSR primer pair markers previously reported for the HGIV locus and mapped more precisely onto the scaffold scf0513158_v1.0 were also included (Isobe *et al*., 2013).

A total of 291 SSR primer pairs used in this study were tested on the reduced sample. Among them, 111 (38%) of the SSR primer pairs failed either to generate a PCR product or did not yield a clear pattern on polyacrylamide gel. The remaining 180 SSR primer pairs generated between one and 15 amplicons with an average of 4.6 products per primer pair. After excluding the monomorphic SSRs, an average of 2.4 alleles was obtained from the polymorphic primer pairs. Actually, if sequences of the primer‐binding sites are conserved in all sub‐genomes, eight homoeologous alleles are expected. The actual number (2.4) is lower, indicating either partial homozygosity or mismatching at primer‐binding sites resulting from sequence divergence in several homoeologous regions.

Out of the 180 SSR primer pairs, 154 SSR primer pairs produced at least one polymorphic band, of which 227 bands were present exclusively in one parent. Each of these 227 bands, considered hereafter as SSR markers, was mapped to one unique bin in either the female (80 SSR markers) or the male (103 SSR markers) parent, whereas the location of the remaining 44 SSR markers could not be determined (Table 2).

**Table 2:** Assignation of the 227 SSR markers to the female or male bins based according to the genetic profile they displayed on the six individuals of the reduced sample and the parents ‘Capitola’ and ‘CF1116’

	Marker number assigned to each bin according to its pattern									
	Female bins					Male bins				
	LGIVa‐f	LGIVb‐f	LGIVc‐f	LGIVd‐f	Not assigned	LGIVa‐m	LGIVb‐m	LGIVc‐m	LGIVd‐m	Not assigned
BIN‐1	13	3	4	2		–	7	–	18	
BIN‐2	–	4	2	14		6	3	3	No bin	
BIN‐3	3	6	2	13		50	No bin	8	No bin	
BIN‐4	No bin	3	6	No bin		No bin	No bin	7	No bin	
BIN‐5	No bin	No bin	5	No bin		No bin	No bin	1	No bin	
Total	16	16	19	29	17	56	10	19	18	27
					97					130

All markers showing a similar pattern for the reduced sample were grouped, and their bin assignment was determined. Among the 80 new markers placed into the female bins, 16, 16, 19 and 29 were respectively attributed to LGIVa‐f, LGIVb‐f, LGIVc‐f and LGIVd‐f (Table 2). Because two of the bins BIN‐FIVb‐3 and BIN‐FIVb‐4 overlapped with the *FaPFRU* locus, the eight SSRs assigned to these two bins (Bx052, Bx056, Bx059, Bx064, Bx083, Bx089, Bx215, Bx250) were further evaluated by mapping them onto the octoploid reference mapping population ‘Capitola’ × ‘CF1116’ (Figure 3; Figure 1, Step4).

The result of mapping these nine markers was in concordance with the bin results, because the six markers of BIN‐FIVb‐3 were located between the markers catc139 and gatt284, and the three markers of BIN‐FIVb‐4 were located between markers gatt284 and gata295 (Figure 3b). Upon the addition of the markers of BIN‐FIVb‐3, LGIVb‐f formed an unique linkage group instead of two distinct ones as previously reported by Gaston *et al*. (2013) (Figure 3a). LGIVb‐f was 125.1 cM long, and the nine markers added to the bottom of this linkage group added an additional 18.7 cM (Figure 3b). Therefore, the use of the bins allowed the drastic reduction in the number of markers to be mapped onto our octoploid reference segregating population and achieved our objective of saturating the *FaPFRU* locus‐containing region with genetic markers.

### Conservation of the *FaPFRU* locus in an independent octoploid population segregating for the PF trait

To determine whether the nine markers are also linked to the PF trait in a genetic background different from ‘Capitola’, we subsequently developed, genotyped and phenotyped a new segregating population from a cross between the PF ‘Mara des Bois’ and SF ‘Pajaro’. Primer pairs Bx089, Bx083, Bx056, Bx059, Bx064 and Bx215 resulted in the amplification of nine polymorphic markers that were mapped onto LGIVb‐f (Figure 3c). Bx052 was monomorphic and Bx250 did not produce an amplicon and so were not mapped. Colinearity of the mapped markers indicated the conservation of this region in both PF cultivars ‘Capitola’ and ‘Mara des Bois’ and suggests that the same introgression event occurred at the *FaPFRU* locus (Figure 3).

### Identification of two markers flanking the *FaPFRU* locus

From the two new maps of ‘Capitola’ and ‘Mara des Bois’, we identified which markers flanked the *FaPFRU* locus using a QTL approach (Figure 1, Step5). Analyses were performed using the total number of inflorescences taken just after emergence that had appeared within the period from the end of May to the end of September, thereby representing the PF trait. This was carried out over 6 and 3 years of observation for ‘Capitola’ × ‘CF1116’ (Gaston *et al*., 2013) and ‘Mara des Bois’ × ‘Pajaro’, respectively. A highly significant QTL (LOD superior to 15 and 10 for the female linkage maps of ‘Capitola’ and ‘Mara des Bois’, respectively) linked to the PF trait was detected on the bottom of LGIVb‐f. The results obtained from ‘Capitola’ allowed us to determine that the maximum values for the QTL were located between the two markers Bx089_196 and Bx064_216 (Figure 3). These two markers were further used for screening larger populations to identify recombinants at the *FaPFRU* locus (Figure 1, Step6).

### Characterization of recombinants reduced the *FaPFRU* locus interval

We screened 1451 individuals issued from different populations (Table 1) for the presence/absence of the two markers, Bx089_196 and Bx064_216, which delimit the *FaPFRU* locus. Out of the 1451 individuals tested (including the individuals of the two mapping populations), 104 recombinants were identified. They displayed only one of the two markers, exhibiting a 0:1 or 1:0 profile (Bx089_196:Bx064_216) (Table 1). Similar percentages of recombinants were observed for each of the segregating population types, derived either from biparental crossing (7%) or by selfing (7%), which were independent of the PF parent, ‘Capitola’ (6%) or ‘Mara des Bois’ (10%). These results suggest that the recombination in this region was stable across different environments and genetic backgrounds.

**Figure 4:**
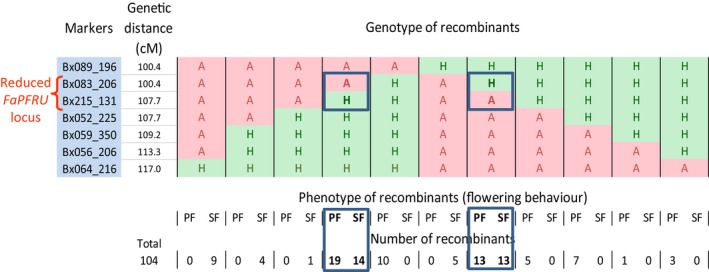
Genotype (H and A for the presence and absence of the markers), phenotype (PF and SF for perpetual and seasonal flowering) and the number of recombinants according to genotype and phenotype. The individuals presenting a recombination (framed in blue) between the markers Bx083_206 and Bx215_131 present both PF and SF behaviours (in bold), indicating that the *FaPFRU* locus controlling the trait of interest is localized between these two markers, which represent the two flanking markers of the reduced *FaPFRU* locus (bracket in red).

To reduce the size of the locus, we genotyped the 104 recombinants with the five SSR markers Bx083_206, Bx215_131, Bx052_225, Bx059_350 and Bx056_206 that are located within the interval framed by the Bx089_196 and Bx064_216 markers (Figure 4; Figure 1, Step7). Combining the five markers, the two frame markers and present (H) or absent (A) controls for each marker, but excluding the two profiles for the absence of recombination, AAAAAAA or HHHHHHH, 12 profiles of recombination could be obtained. Of the 104 recombinants, only the AAAAAHH profile was not observed. Observing 11 out of 12 possible profiles indicated that recombination events at the *FaPFRU* locus are well represented (Figure 4 and Table S2). By adding the phenotypic value of the trait to the locus, PF or SF, we were able to assign the *FaPFRU* locus to the interval between the Bx083_206 and Bx215_131 markers, which were 7.3 cM apart in the ‘Capitola’ × ‘CF1116’ reference map.

### Identification of the *FLOWERING LOCUS T (FaFT)* gene as a promising candidate controlling the PF trait

**Figure 5:**
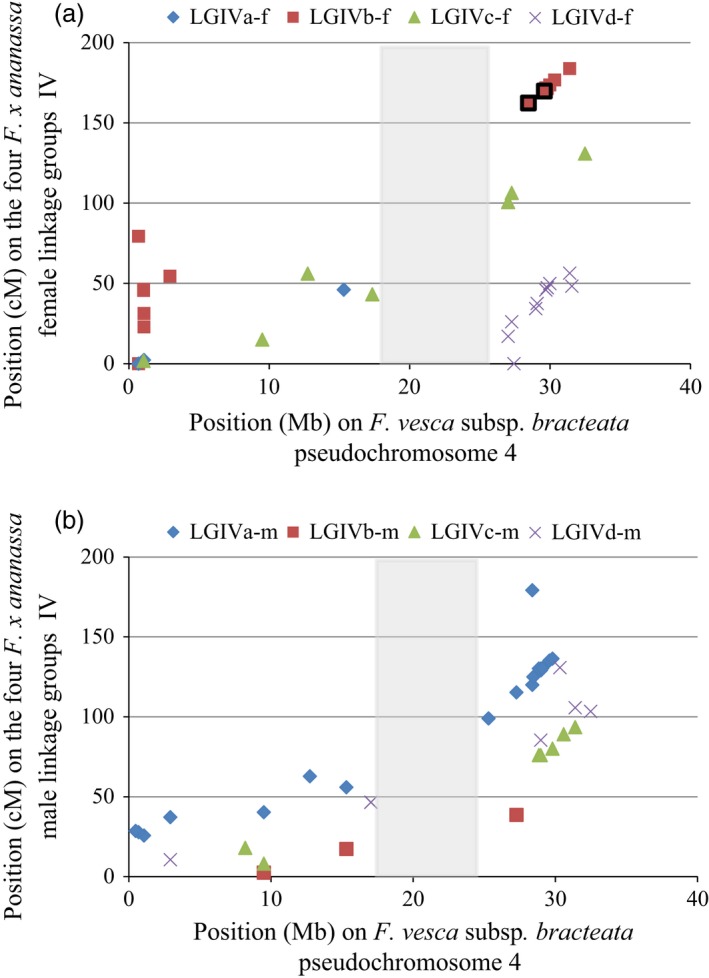
Comparison between physical and genetic distances for all informative markers (SSRs) localized on the four homoeologous (a) female and (b) male linkage groups (LG) IV. Physical distance was identified thanks to the pseudochromosome‐4 sequence of the *F. vesca* reference genome v2.0.a1. Genetic distances are identified thanks to linkage maps of the octoploid population ‘Capitola’ × ‘CF1116’. The two flanking markers of the reduced *FaPFRU* locus on the LGIVb‐f are framed in black. In grey, genomic region without SSR markers in our study.

As a prelude to a detailed investigation into the region corresponding to the reduced *FaPFRU* locus interval for the diploid reference genome, we compared for each HGIV of the cultivated strawberry its genetic distance in cM with the physical distance of the corresponding LGIV in the diploid reference genome v2.0.a1 (available on www.rosaceae.org) (Jung *et al*., 2014; Tennessen *et al*., 2014). For each linkage group of HGIV, the markers are evenly distributed along a line (Figure 5) revealing a well conserved genetic organisation between the diploid reference genome and the octoploid genetic map for each of the HGs and the presence of a region without SSR markers between 17.3 Mb and 25.3 Mb. This gap may have been due to the presence of exclusively homozygous sequences or to substantial rearrangements between the diploid and the octoploid genomes in this region. However, previously mapped AFLP markers (Lerceteau‐Köhler *et al*., 2003), which were based on arbitrary sequences and could not be placed in the diploid reference genome, successfully revealed polymorphism in this region. These results support the hypothesis of a major DNA rearrangement rather than the consequence of homozygosity.

Based on the diploid reference genome v2.0.a1, the region between markers Bx083_206 and Bx215_131 flanking the *FaPFRU* locus is 1108 Kb and contains 234 predicted genes. An analysis of gene ontology (GO) was performed to assign possible functions to each of the 234 genes. Out of the 234 genes, 72 yielded no hit in the SwissProt database and were not annotated, but 15 were classified as flowering associated (Table 3). Among these 15 genes, the most likely candidate for the control of PF was a gene homologous to *FT*, the *gene04680* (Gaston *et al*., 2013).

**Table 3:** Gene Ontology (GO) for the 15 candidate genes linked to flowering and present in the reduced interval of the *FaPFRU* locus delimited by Bx083 and Bx215 SSRs (E values superior to 10^−5^)

Name	Annotation	Gene fonction
*gene04680*	Flowering locus T (FT)	Florigen
*gene04683*	Protein Argonaute 7 (AGO7)	Gene silencing by miRNA
*gene04726*	Agamous‐like Mads‐box protein (AGL30)	Member of the MIKC family of transcriptional regulators
*gene04596*	Leucine‐Rich Repeat Receptor like kinase (RPK2)	Regulator of meristem maintenance
*gene03989*	Sucrose transport protein (SUC3)	Sucrose transport
*gene04730*	Cyclic dof factor 2 – zinc finger protein (CDF2)	DNA binding transcription factor activity
*gene04745*	Zinc finger homeodomain protein 9 (ZHD9)	DNA binding transcription factor activity
*gene03991*	Homeobox leucine zipper protein (ATHB‐13)	DNA binding transcription factor activity
*gene04031*	Homeobox‐leucine zipper protein (HAT5)	DNA binding transcription factor activity
*gene04729*	Probable calcium‐binding protein 45 (CLP45)	Involved in calcium transport
*gene04028*	Cullin‐1 (CUL1)	Involved in ubiquitination process
*gene04757*	Cullin‐3a (CUL3a)	Involved in ubiquitination process
*gene04002*	Ubiquitin‐conjugating enzyme E2 (UBC2)	Involved in ubiquitination process
*gene04611*	Ureide Permease 2 (UPS2)	Uracyl transport
*gene04612*	Ureide Permease 2 (UPS2)	Uracyl transport

### Effect of the *FaPFRU* locus on the plant development

**Figure 6:**
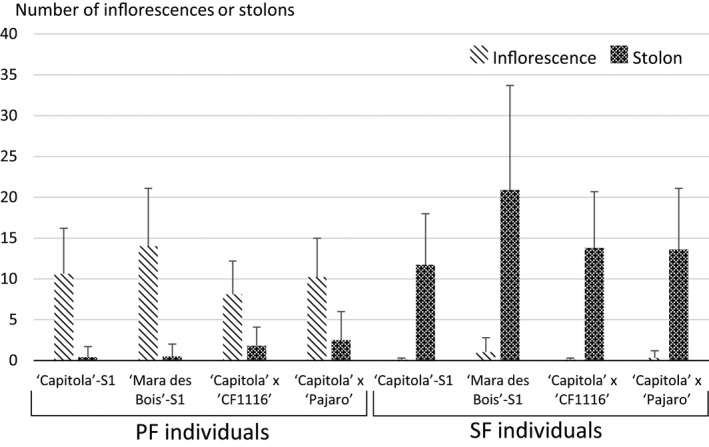
The number of inflorescences and stolons of individuals belonging to four populations scored in 2012 according to their flowering behaviour, perpetual flowering (PF) or seasonal flowering (SF).

An outstanding question regarding the *FaPFRU* locus is its influence on other plant developmental traits. One trait of interest is the relationship between the number of inflorescences and stolons produced by a plant, which was addressed by scoring the number of inflorescences and stolons during the period from the end of May until the end of September in a subset of four populations segregating for this trait, ‘Capitola’‐S_1_, ‘Mara des Bois’‐S_1_, ‘Capitola’ × ‘CF1116’ and ‘Capitola’ × ‘Pajaro’. In agreement with previous findings in Gaston *et al*. (2013), we determined that PF individuals generated 4–28 times more inflorescences than stolons, whereas SF individuals produced a large number of stolons, but almost no inflorescences (Figure 6). These results revealed the opposite effects of *FaPFRU* on flowering (positive) and runnering (negative) independent of genetic background.

**Figure 7:**
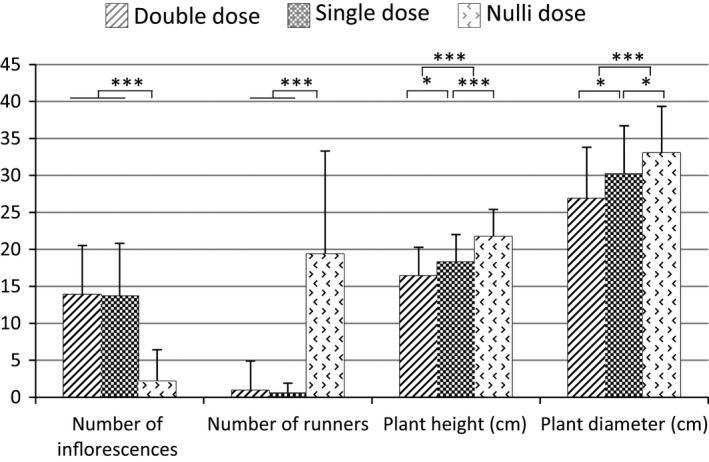
Dose effect of the co‐dominant markers Bx089_215c/Bx089_220r localized in the *FaPFRU* locus. Phenotyping was performed on ‘Mara des Bois’‐S_1_ population. Double Dose indicates homozygous individuals for Bx089_215c (linked in coupling to the *FaPFRU* allelic variant), Single Dose indicates heterozygous individuals for Bx089_215c/Bx089_220r and Nulli Dose indicates homozygous individuals for Bx089_220r. Asterisks indicate significant differences by Student's t‐test: *, **, *** for statistical significance at *P* ≤ 0.05, 0.01, 0.001, respectively.

The *FaPFRU* locus also negatively affected plant size. Using co‐dominant markers, in particular Bx089_215c/Bx089_220r, we investigated the dose effect of these markers on plant height and diameter in the ‘Mara des Bois’‐S_1_ population. In SF individuals showing homozygosity of the marker Bx089_220r (or Null Dose (ND) of Bx089_215c), the plants displayed the largest height and diameter. Among PF individuals, heterozygous plants (Bx089_215c/Bx089_220r) were taller and larger than homozygous plants (Bx089_215c in Double Dose, DD) (Figure 7). However, no difference between homozygous and heterozygous PF individuals was observed in the number of either inflorescences or stolons. The negative effect on plant height was confirmed by the identification of a QTL located at the *FaPFRU* locus, which was linked to the height measurements recorded in 2003 and 2004 (maximum LOD values of 7.1 and 5.9, respectively). For both years, the decrease in plant height was around 3 cm for PF individuals compared to that for SF individuals.

### Additional validation using genetic resources

The presence or absence of the two markers Bx083_206 and Bx215_131 flanking the *FaPFRU* locus was investigated in 79 cultivars or selections of *F*. × *ananassa* (Table 4 and Table S3). The absence of both markers observed for 47 SF cultivars or selections was strictly associated with SF genotypes. The presence of both markers flanking the *FaPFRU* locus was associated with the PF trait for 14 (82%) out of the 17 PF cultivars/selections. However, nine out of the 62 SF cultivars/selections displayed both markers and six cultivars showed recombination in this region by displaying one of the two flanking markers. This study demonstrated that these markers will be useful in breeding programmes, independently of parental display of either or both flanking markers. It further reinforce the hypothesis that a single allelic variant controlling PF has been maintained through breeding of modern strawberry cultivars.

**Table 4:** Distribution of the 79 cultivars/selections belonging to genetic resources according to their status on the two flanking markers

Flowering behaviour of genotypes	Marker status Bx083_206/Bx215_131			Total
	+/+	+/− or −/+	−/−	
Seasonal flowering	9	6	47	62
Perpetual flowering	14	3	0	17

## Discussion

The perpetual flowering (PF) trait of strawberry extends the length of the flowering period and so can prolong the fruit production season. Thus, it contributes not only to improve fruit yield but also to open new markets for strawberries, for example by offering fruit to consumers outside the production season. For these reasons, most strawberry breeding programmes have aimed at introgressing this trait into elite cultivars. However, this task remains challenging and requires considerable effort, due to the interaction of the PF trait with its genetic and environmental context (Maltoni *et al*., 1996). The identification of genetic markers in close linkage to the *FaPFRU* locus will permit the discrimination of PF cultivars/ selections at very early stages of plant development, thereby circumventing the need for phenotyping the large numbers of mature plants under favourable environment conditions. Identification of the *FaPFRU* allelic variant will further provide insights into the mechanisms governing the balance between flowering and runnering in strawberry.

### A strategy to narrow down the *FaPFRU* locus that circumvents polyploid complexity

The *FaPFRU* locus found in the cultivated strawberry results from the introgression of a PF locus from a *F. virginiana* ssp. *glauca* Wasatch accession collected from the Wasatch Mountains in Utah (Bringhurst and Voth, 1984; Powers, 1954). From then on, this original introgression has been largely used in breeding until today (Hancock, 1999). However, developing markers to follow this trait has been largely impeded by the polyploid status of the cultivated octoploidy strawberry.

With recent and ancestral events of whole‐genome duplication (WGD) events, nearly all seed plants have experienced episodes of polyploidy (Jiao *et al*., 2011; Renny‐Byfield and Wendel, 2014). In diploid species, such as *Arabidopsis thaliana*, successive events of duplications have led to the colinearity of sections of the genome (Blanc *et al*., 2000). Duplication of genomes complicates mapping of markers for several reasons. It requires substantial effort to find polymorphisms between homoeologous regions in closely related sub‐genomes (Njuguna *et al*., 2013) and to decipher the effect of multi‐dose markers (van Dijk *et al*., 2014). Furthermore, the divergence among homoeologs could be influenced by dynamic genomic changes revealing asymmetrical evolution of sub‐genomes (Liu *et al*., 2014) or affecting many sites at once by introgression and chromosomal rearrangements (Tennessen *et al*., 2014). Most mapping strategies require considerable work to circumvent these problems in polyploid crop species, and so map‐based breeding remains very costly. In recent years, highthroughput genotyping of complex polyploid species has benefited considerably from improved genetic linkage maps possessing greater marker density. Nevertheless, gaps are still present as shown in the work using SNP arrays in strawberry (Bassil *et al*., 2015) and cotton (Hulse‐Kemp *et al*., 2015) or through DArT markers in strawberry (Sánchez‐Sevilla *et al*., 2015) or *Brassica* (Raman *et al*., 2013). Within these linkage maps, marker coverage may be uneven and/or sparse in some genomic regions due to the lower polymorphism level in some sub‐genomes, as shown for wheat (Maccaferri *et al*., 2015), cotton (Blenda *et al*., 2012) and strawberry (Tennessen *et al*., 2014). Consequently, a unique LG may become split into different sub‐LGs, as observed for the strawberry LGIVb‐f that has been separated into two sub‐LGs (Gaston *et al*., 2013).

In strawberry, one genome of the diploid species corresponds to four homoeologous genomes in the cultivated octoploid species (Rousseau‐Gueutin *et al*., 2009; Tennessen *et al*., 2014). In this context, the fine mapping of a specific homoeologous region by marker enrichment may prove unrevealing or, at best, very time consuming. For example, markers suffer from random mapping to one of four sub‐genomes instead of being mapped to the specific sub‐genome carrying the homoeologous region of interest. We reasoned that the selective mapping strategy first described by Vision *et al*. (2000), which was successfully used in diploid crop species (e.g. Cabrera *et al*., 2009; Howad *et al.,* 2005; Sargent *et al*., 2008), could be applied to complex polyploid species to overcome these limitations. When applied to polyploids, this strategy employs a reduced sample of highly informative individuals chosen according to the position of their recombinational crossover sites in the homoeologous region of interest. In strawberry, it facilitated the joining of the two sub‐linkage groups of LGIVb‐f into a unique LG and helped to dramatically narrow down the single homoeologous *FaPFRU* locus carrying the allelic variant controlling the PF trait. As a consequence, flanking markers tightly linked to *FaPFRU* are available for MAS, and the candidate genes comprising the locus have been identified. This locus is likely the same as the one described in previous studies (Castro *et al*., 2015; Honjo *et al*., 2016) because all markers developed in our work and in previous studies are located on the same chromosomal region of the LG4 in the diploid reference genome. However, the flanking markers developed in our study are closer to the locus as shown in Figure S1.

This strategy remains relevant even with more dense linkage maps, which still show gaps.

If a more saturated map is used instead of a framework linkage map, the overall strategy will be the same, but smaller bins will be obtained. In addition, when taking into account multi‐dose markers useful for the identification of homoeoQTLs (Lerceteau‐Köhler *et al*., 2012), the choice of an appropriate reduced sample of individuals should allow the fine mapping of different homoeologous loci.

### PF has a single origin in cultivated strawberry and has a different genetic control than in diploid strawberry

In addition to the *FaPFRU* locus that originated from the *F. virginiana* species (Bringhurst and Voth, 1984; Powers, 1954), two other origins of PF trait in the cultivated strawberry have been reported (Darrow, 1966). The cultivar ‘Pan American’ released in the USA in 1902 is believed to be a mutant of the Bismarck cultivar, whereas Gloede's seedling was first identified in France (Richardson, 1914). Thus, there is still a doubt regarding the origin of the PF trait in modern cultivated cultivars. In this study, we tested 79 different cultivars and selections from our strawberry germplasm, including 17 PF cultivars from various breeding programmes from around Europe, Japan and USA. Results showed the presence of at least one of the flanking markers of the *FaPFRU* locus within the various PF cultivars, confirming that the modern PF cultivars have been derived from the same *FaPFRU* introgression event. Because of time inconsistency between the release in Europe of the PF parent of ‘Mara des Bois’ (‘Hummi Gento’ 1967) and the first release of modern PF cultivars related to ‘Capitola’ in USA (1971–1972) (Bringhurst and Voth, 1980), it had been previously assumed that the origin of the PF behaviour in these cultivars was different. However, we identified in this study six SSR markers in linkage disequilibrium with the PF allele that were identical in ‘Mara des Bois’ and ‘Capitola’. It is likely that lines selected from offsprings from the Wasatch genotype were sent from USA to Europe before the release of the first PF cultivars in USA. We therefore believe that the origin of the PF allele is the same in ‘Mara des Bois’ and ‘Capitola’.

In addition, we found that PF cultivars in the tested germplasm are heterozygous at this locus (data not shown), suggesting that the presence of a dominant homozygous allelic variant may be a disadvantage. In fact, a significant negative co‐dominant effect of an *FaPFRU* allelic variant on plant size was observed, indicating that the *FaPFRU* locus plays a major role in strawberry plant fitness by controlling the balance between sexual reproduction and vegetative development independent of dose. Although this effect was not linked to the number of inflorescences produced by the plant, a trade‐off between fruit production and growth of vegetative organs is possible, such as through competition for available carbon.

The diploid and octoploid PF strawberries share similar physiological control of the perpetual flowering process (Sønsteby and Heide, 2008). PF occurs during long days and is antagonistic to the stolon production (Tenreira T and Denoyes B, unpublished data). Deciphering the mechanisms involved in the regulation of PF and runnering in strawberry through the identification of allelic variants underlying the variations in both traits will be of considerable help. Genetic controls of known PF traits in diploid and cultivated octoploid strawberry are likely to be different. PF was mapped to LGVI in diploid strawberry *F. vesca* (Albani *et al*., 2004) and to LGIVb in octoploid cultivated strawberry *F. × ananassa* (Gaston *et al*., 2013). We and others previously showed that a two‐bp mutation in *FvKSN,* a strawberry ortholog of the *TERMINAL FLOWER 1* (*TFL1*) floral repressor, was responsible for the PF trait in diploid strawberry (Iwata *et al*., 2012; Koskela *et al*., 2012). The role of *TFL1* as a floral repressor in the cultivated octoploid strawberry was also recently confirmed by Koskela *et al*. (2016). In this study, silencing the floral repressor *FaTFL1* in a SF cultivar through a transgenic approach was sufficient to induce PF under long days. In the cultivated octoploid strawberry, Nakano *et al*. (2015) proposed that *FaFT3*, one of the three *FT‐like* genes described in *Fragaria* (Iwata *et al*., 2012), is a key gene in flower induction. In a recent study using *F. vesca*, Mouhu *et al*. (2013) further explored the possible implication of orthologs of *SUPPRESSOR OF OVEREXPRESSION OF CONSTANS 1* (*FvSOC1*) and *FLOWERING LOCUS T* (*FvFT1*), which are two genes upstream of *KSN/TFL1* in the flowering signalling pathway. Inactivation of *FvSOC1* through RNAi‐silencing led to PF. However, evidence is lacking that *FvFT1* inactivation would lead to PF in a SF genetic background. The patterns of *FvFT1* expression suggest that it may influence the photoperiodic regulation of *FvSOC1* (Koskela *et al*., 2012; Mouhu *et al*., 2013). Although *FaTFL1*,* FaFT3*,* FaFT1* and *FaSOC1* appear as potential candidates to regulate PF in cultivated octoploid strawberry, they can be excluded because *FvSOC1* is located on LGVII, *FvFT3* is located on LGIII and *FvTFL1* and *FvFT1* are located on LGVI in diploid strawberry. In contrast, the *FaFT‐like* gene (named *FaFT2* in Nakano *et al*., 2015) that is orthologous to *gene04680‐v1.0‐hybrid*, and which is located within the reduced *FaPFRU* locus, appears to be an excellent candidate. Like *TFL1*,* FT* belongs to the phosphatidylethanolamin binding protein (PEBP)‐encoding gene family and plays a crucial role in flowering. Whereas TFL1 protein represses the transition from vegetative to reproductive phase, FT protein promotes these phases and is considered to be the florigen (Corbesier *et al*., 2007). FT and TFL1 proteins may compete for binding to the transcription factor FD to either activate or repress the meristem identity genes (Hanano and Goto, 2011; Pin and Nilsson, 2012; review in Wickland and Hanzawa, 2015). In this hypothesis, an allelic variant of *FaFT2* would act as a positive regulator of flowering. *FaFT2* would therefore overcome the floral repression in long days carried by *FaTFL1* in PF‐cultivated octoploid strawberry. Although *FaFT‐like* is the most likely candidate, the other known flowering associated genes should not be neglected in future studies aimed at deciphering the PF‐regulating mechanisms in cultivated strawberry.

## Conclusion

Perpetual flowering is as a major agronomical trait, because its characteristics permit the extension of the fruit production season. The development of an original approach, based on selective mapping to enrich a single region of one specific homoeologous linkage group, allowed us to fine map the *FaPFRU* locus controlling PF in the highly polyploid cultivated strawberry. We described the rapid development of two flanking markers, which were subsequently validated in a second segregating population and in genetic resources. These findings lay the foundation for more rapid and efficient creation of new PF strawberry cultivars through the marker‐assisted selection. In addition, the identification of likely candidate genes will advance our understanding of flowering in strawberry.

## Experimental procedures

### Plant materials and PF phenotyping

Plant material of the cultivated octoploid strawberry, *F. × ananassa*, which was used for fine mapping the region controlling the PF trait and enhancing the prevalence of crossover events in this region, was obtained from various crosses or selfings. These populations were issued from the heterozygous PF parents ‘Capitola’ or ‘Mara des Bois’ and the homozygous SF parents ‘CF1116’ or ‘Pajaro’ (Table 1). For the two pseudo, full‐sibling F_1_ populations ‘Capitola’ × ‘CF1116’ and ‘Mara des Bois’ × ‘Pajaro’ linkage maps were created. The first one representing our reference segregating population to identify the *FaPFRU* locus (Gaston *et al*., 2013) was increased to 375 individuals for this study to facilitate identifying recombinants. For the second population, a framework linkage map was obtained from 85 individuals. Recombinants were screened in these two populations described above and 212 individuals of a third F_1_ population ‘Capitola’ × ‘Pajaro’, and selfings of four genotypes ‘Capitola’‐S_1_, ‘Mara des Bois’‐S_1_, [‘Mara des Bois’ × ‘Pajaro’]‐F_2_‐3 and [‘Mara des Bois’ × ‘Pajaro’]‐F_2_‐45 that were already known to be heterozygous for the PF trait. All together, these various populations provided 1451 F_1_, S_1_ or F_2_ individuals.

Seeds were germinated in the greenhouse with a 16‐h photoperiod using supplementary incandescent light and a day and night temperatures of 24 ± 6 °C and 18 ± 4 °C, respectively. Four to six weeks after sowing, seedlings were transplanted to 72‐cell plug trays containing sand/vermiculite substrate (1:1 by volume) at a density of one plant per 5 × 5 cm well.

For studying the presence of markers linked to the PF behaviour in the genetic resources, we analysed 79 cultivars or selections with various pedigrees and origins (Table S3).

Flowering behaviour of individuals of the two mapping populations and recombinants was identified as described in the study by Gaston *et al*. (2013). In addition, selfings of ‘Mara des Bois’‐S_1_ were characterized for their plant size, height and diameter.

For QTL analyses, phenotypic data were represented by the number of newly‐emerged inflorescences from the end of May to the beginning of August and were described in the study by Gaston *et al*. (2013) or obtained after 3 years of observation for ‘Capitola’ × ‘CF1116’ and ‘Mara des Bois’ × ‘Pajaro’ populations.

### DNA extraction

Genomic DNA was extracted using the DNeasy^™^ Plant Mini Kit or DNeasy^™^ 96 Plant Kit (Qiagen Inc., Hilden, Germany) with 90 mg or 45 mg, respectively, of young, developed, leaf tissue according to the manufacturer's protocol and modified as previously described (Lerceteau‐Köhler *et al*., 2003). gDNA was quantified visually using the DNA‐binding dye GelGreen^™^ (Biotium, Hayward, CA) and standards of known quantity.

### Reduced sample choice for optimizing the identification of markers on the LGIVb‐f

A goal was to reduce the effort required to obtain new markers in coupling‐phase linkage with the PF‐specific allele at *FaPFRU*, rather than the corresponding, SF‐specific allele. Therefore, we developed a selective mapping procedure based on the selection of a specific set of highly informative individuals chosen for the position of their recombinational crossover sites (Vision *et al*., 2000) in the homoeologous group IV, in which the LGIVb‐f carries the allelic variant *FaPFRU*. The reduced set was a subset of plants from the ‘Capitola’ × ‘CF1116’ pseudo full‐sibling F_1_ population, for which framework female and male (f and m) maps were previously obtained (Lerceteau‐Köhler *et al*., 2003; Rousseau‐Gueutin *et al*., 2008). BIN names consisted of BIN, the female or male map, the LG, and a number indicating the relative position of the bin on the LG. For example, BIN‐MIVb‐2 indicates BIN 2 on the male LGIVb.

### Development of SSR markers for fine mapping

Simple sequence repeat primer pairs for fine mapping were designed from the *F. vesca* v2.0.a1 Pseudomolecule Assembly (Tennessen *et al*., 2014) using colinearity with the homoeologous genomes of the cultivated strawberry. We focused on the large scaffold scf0513158_v1.0 (4 198 144 bp) located at LGIVb‐f, which was upstream from the UDF008 marker at the end of the *FaPFRU* QTL (Gaston *et al*., 2013) (Figure 3). SSR motifs were detected by the Phobos plug‐in compatible with the Geneious software (version 6.1.4; Biomatters Ltd, Auckland, New Zealand). Primers were designed for repeats that contained at least 10 bp or for combinations of repeats. See Table S4 for the primers used in this study. Primers were designed in the SSR flanking regions based on the diploid reference genome using Primer3 plug‐in of Geneious. In addition, SSR primer pairs previously published and located on the HGIV, and more precisely on the scaffold scf0513158_v1.0, were also studied (Isobe *et al*., 2013).

PCR was performed using the standard PCR protocols at an annealing temperature of 55 °C. Amplicons derived from the 291 SSRs obtained on the reduced sample were screened on 6% (w/v) polyacrylamide gels as previously described (Rousseau‐Gueutin *et al*., 2008). The seven markers tested on recombinants were formed on the basis of screening all 1451 individuals using a specific PCR protocol, and amplicons were visualized using an Applied Biosystems (ABI) 3730 DNA Analyzer (Foster City, CA, USA) to obtain allele sizes. Cycling conditions for all primers except Bx215 were as follows: an initial denaturation at 94 °C for 3 min followed by 30 cycles of 94 °C for 40 s, 55 °C for 45 s and 72 °C for 45 s. Primer pair Bx215 was annealed at 58 °C. These cycles were followed by eight cycles of PCR with a lower annealing temperature to incorporate the 6‐FAM^™^ M13 or VIC^®^ M13 label at 94 °C for 40 s, 53 °C for 45 s and 72 °C for 45 s, with a final extension at 72 °C for 30 min. PCR products were diluted from 1:20–1:50 according to the primer pairs for the fragment analysis on an ABI 3730 DNA Analyzer calibrated with an ABI Genescan^™^ 600 LIZ Size Standard (reference 4366589). Peaks data were scored using GeneMapper V4.0 software (Applied Biosystems, Foster City, CA, USA) and converted to a qualitative, binary code to facilitate scoring for either the presence or absence. For each SSR marker, the first part of the identifier indicates the name of the SSR followed by a number indicating the allele size in bp. For marker Bx064_122 for example, Bx064 states the SSR primer pairs, and 122 represents the allele size of the amplicon.

### Development of markers that flanked the QTL for further recombinant identification

For QTL detection on the female linkage map of ‘Capitola’ × ‘CF1116’, we used the framework map previously developed (Lerceteau‐Köhler *et al*., 2012; Rousseau‐Gueutin *et al*., 2008), including the nine markers at LGIVb‐f found in this study, and the phenotypic data were those described in the study by Gaston *et al*. (2013). QTL analyses were performed by the composite interval mapping (CIM) (Zeng, 1993, 1994) using QTL Cartographer software (Basten *et al*., 1997) as described in the study by Gaston *et al*. (2013). For ‘Mara des Bois’ × ‘Pajaro’, similar QTL analyses were performed on the seven linkage groups constructed with 37 markers. This linkage map included the orthologous LGIVb.

The QTL analysis yielded the two flanking alleles delimiting the peak of the *FaPFRU* locus that were used for the subsequent identification of recombinants (Figure 3). The 1,451 S_1_ or F_2_ seedlings were amplified with primer pairs of Bx083 and Bx215 for genotyping the two flanking alleles Bx083_206 and Bx215_131. Only recombinants showing one of the two markers were kept for further phenotyping.

### Comparison between genetic and physical distances in the HGIV

To see whether the mapped markers were distributed regularly on the homoeologous chromosomes belonging to the HGIV, we compared genetic with physical distances. This comparison was performed on both obtained female and male linkage groups with the pseudomolecule IV of the diploid reference genome (v2.0.a1). For identifying the physical map location of the microsatellite markers used in the genetic octoploid map, we used BLAST against the *F. vesca* v2.0.a1 Pseudomolecule Assembly: (Tennessen *et al*., 2014). Genetic distances were obtained using the software MapMaker/Exp v3.0 (Lander *et al*., 1987).

### Identifying candidate genes within the refined region

Within the interval framed by the SSRs Bx083_206 and Bx215_131, a gene search was made in GDR browser on the hybrid version of the genome annotation (http://www.rosaceae.org). The proteins of the identified genes were subjected to a Blast2Go analysis (Conesa *et al*., 2005) to predict function through gene ontology (GO). A BLASTp was run against the SwissProt database with an E value at 10^−5^. The mapping and annotation steps were run using default parameters. Genes were filtered for those predicted to be involved in the biological processes related to flowering.

## Supporting information


**Figure S1** Position on the *F. vesca* subsp. *bracteata* pseudochromosome 4 (Tennessen *et al*., 2014) of the markers linked to perpetual flowering in the octoploid strawberry developed in this study (in bold), in Castro *et al*. (2015) and in Honjo *et al*. (2016) (in italic).

**Table S1** Description of the 15 and 11 female and male bins of the four linkage groups (LGs) IV in the octoploid *Fragaria* reference map.
**Table S2** Number of recombinants according to their population of origin, genotype and phenotype. Individuals were fine mapped between Bx089_196 and Bx064_216 SSRs. Phenotype was recorded as Perpetual Flowering (PF) or Seasonal Flowering (SF).
**Table S3** List of 79 cultivars or selections tested for presence (1) or absence (0) of two SSR markers that flanked the *FaPFRU* locus. Pedigree, year of cultivar release, country of origin, and year of sampling are included.
**Table S4** List of the SSR primer pairs used in this study.
