# Supplementary material for: Narrowing down the single homoeologous FaPFRU locus controlling flowering in cultivated octoploid strawberry using a selective mapping strategy
Source: Plant Biotechnol J. 2016 Jun 9;14(11):2176–89. doi: 10.1111/pbi.12574 (PMC5095798; doi:10.1111/pbi.12574)
Supplement: Supplementary file 1 — Figure S1 Position on the F. vesca subsp. bracteata pseudochromosome 4 (Tennessen et al., 2014) of the markers linked to perpetual flowering in the octoploid strawberry developed in this study (in bold), in Castro et al. (2015) and in Honjo et al. (2016) (in italic). [file PBI-14-2176-s002.docx]

^
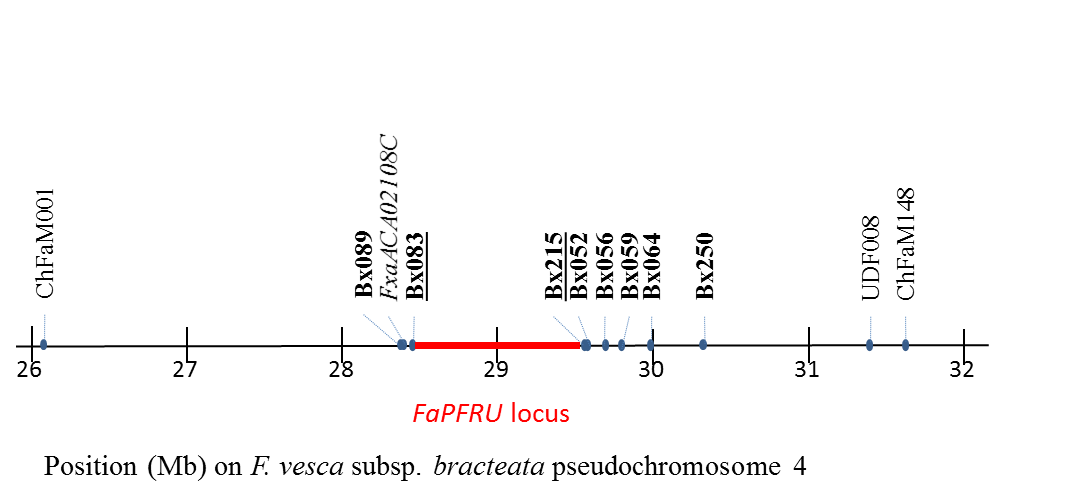
^

**Supplemental Figure 1.** Position on the *F. vesca* subsp. *bracteata* pseudochromosome 4 (Tennessen *et al*., 2014) of the markers linked to perpetual flowering in the octoploid strawberry developed in this study (in bold), in Castro *et al*. (2015) and in Honjo *et al.* (2016) (in italic). The position of the *FaPFRU* locus is indicated in red and the flanking markers are underlined.
