# Supplementary material for: Narrowing down the single homoeologous FaPFRU locus controlling flowering in cultivated octoploid strawberry using a selective mapping strategy
Source: Plant Biotechnol J. 2016 Jun 9;14(11):2176–89. doi: 10.1111/pbi.12574 (PMC5095798; doi:10.1111/pbi.12574)
Supplement: Supplementary file 2 — Table S1 Description of the 15 and 11 female and male bins of the four linkage groups (LGs) IV in the octoploid Fragaria reference map. Table S2 Number of recombinants according to their population of origin, genotype and phenotype. Individuals were fine mapped between Bx089_196 and Bx064_216 SSRs. Phenotype was recorded as Perpetual Flowering (PF) or Seasonal Flowering (SF). Table S3 List of 79 cultivars or selections tested for presence (1) or absence (0) of two SSR markers that flanked the FaPFRU locus. Pedigree, year of cultivar release, country of origin, and year of sampling are included. Table S4 List of the SSR primer pairs used in this study. [file PBI-14-2176-s001.docx]

**Supplemental Table 1.** Description of the 15 and 11 female and male bins of the four linkage groups (LGs) IV in the octoploid *Fragaria* reference map. These bins were identified with a reduced sample of six individuals in addition to the two parents, ‘Capitola’ and ‘CF1116’.

| Linkage group | | Genotype of the six individuals of the reduced sample | Characteristics of the bins | | |  | No. of markers | |
| --- | --- | --- | --- | --- | --- | --- | --- | --- |
|  |  |  | Bin name | Starting marker | Ending marker | Interval (cM) | Initial | New markers |
| **Female bins** | |  |  |  |  |  |  |  |
| LGIVa-f |  | HHAAAA | BIN-FIVa-1 | gtag268c | tggx136c | 6.3 | 4 | 13 |
|  |  | HHHAAA | BIN-FIVa-2 | tggx184c | i175199r | 15.5 | 5 | - |
|  |  | AHAAHA | BIN-FIVa-3 | tcag310c | v007184c | 2.1 | 3 | 3 |
| LGIVb-f | LGIVb1-f | HAHHAA | BIN-FIVb-1 | tgat134r | ccaa410c | 32.1 | 7 | 3 |
|  |  | HAHHHA | BIN-FIVb-2 | catc139c | catc139c | 0 | 1 | 4 |
|  | LGIVb-f2 | AAHAAH | BIN-FIVb-3 | gatt284c | gatt284c | 0 | 1 | 6 |
|  |  | AHAAAH | BIN-FIVb-4 | gata295c | u008123c | 3.1 | 2 | 3 |
| LGIVc-f |  | HAHHAH | BIN-FIVc-1 | gatc143r | tcac210s | 9.1 | 3 | 4 |
|  |  | HAAHHH | BIN-FIVc-2 | catc184c | i175202c | 6.9 | 4 | 2 |
|  |  | AAAHHH | BIN-FIVc-3 | gata185r | tcag088c | 35.3 | 7 | 2 |
|  |  | AAAHAA | BIN-FIVc-4 | b008168r | b008168r | 0 | 1 | 6 |
|  |  | AAHHAA | BIN-FIVc-5 | tggx147c | tggx147c | 0 | 1 | 5 |
| LGIVd-f | LGIVd1-f | AHHAHH | BIN-FIVd-1 | gttt218c | gttc187c | 35 | 6 | 2 |
|  | LGIVd2-f | HAAAHA | BIN-FIVd-2 | i136147c | gtgx440c | 11.2 | 2 | 14 |
|  |  | HHHAHA | BIN-FIVd-3 | tgtg253c | gaag490c | 10.9 | 4 | 13 |
| *Sub-Total* |  |  |  |  |  |  | *51* | *80* |
|  |  |  |  |  |  |  |  |  |
| **Male bins** | |  |  |  |  |  |  |  |
| LGIVa-m |  | HHAAHA | BIN-MIVa-1 | gatc380 | c014162 | 30 | 10 | - |
|  |  | AAAAHA | BIN-MIVa-2 | b008145 | M014164 | 54 | 16 | 6 |
|  |  | HHAAHA | BIN-MIVa-3 | tggx350 | gtta105 | 31.6 | 6 | 50 |
| LGIVb-m |  | AAAAAH | BIN-MIVb-1 | gatt252 | gatt298 | 43 | 13 | 7 |
|  |  | AAAAHH | BIN-MIVb-2 | tcaa317 | ccaa220 | 11.2 | 4 | 3 |
| LGIVc-m |  | HHAAHA | BIN-MIVc-1 | gtat238 | gtat238 | 0 | 1 | - |
|  |  | HHAHHA | BIN-MIVc-2 | b008165 | tggx318 | 21.8 | 7 | 3 |
|  |  | HHAAHA | BIN-MIVc-3 | tgat165 | gtta242 | 24.9 | 3 | 8 |
|  |  | HHHAHH | BIN-MIVc-4 | gaac250 | gaac250 | 13.2 | 1 | 7 |
|  |  | HHHAAH | BIN-MIVc-5 | tgtg215 | tgtc328 | 41.7 | 5 | 1 |
| LGIVd-m | LGIVd1-m | AAAHAA | BIN-MIVd-1 | tgag400 | u008113 | 98.7 | 15 | 18 |
| *Sub-Total* |  |  |  |  |  |  | *81* | *103* |

**Supplemental Table 2.** Number of recombinants according to their population of origin, genotype and phenotype. Individuals were fine mapped between Bx089_196 and Bx064_216 SSRs. Phenotype was recorded as Perpetual Flowering (PF) or Seasonal Flowering (SF).

| SSR markers ^(a)^ | | | | | | | ‘Capitola’ x ‘CF1116’ | | | ‘Capitola’ x ‘Pajaro’ | | | ‘MdB’^(b)^ x ‘Pajaro’ | | | ‘Capitola’ | | | ‘Mara des Bois’ | | | [‘MdB’ x ‘Pajaro’]F_1_-n°45 | | | [‘MdB’ x ‘Pajaro’]F_1_-n°3 | | |  |
| --- | --- | --- | --- | --- | --- | --- | --- | --- | --- | --- | --- | --- | --- | --- | --- | --- | --- | --- | --- | --- | --- | --- | --- | --- | --- | --- | --- | --- |
|  |  |  |  |  |  |  |  |  |  |  |  |  |  |  |  |  |  |  |  |  |  |  |  |  |  |  |  |  |
| Bx089_196 | Bx083_206 | Bx215_131 | Bx052_225 | Bx059_350 | Bx056_206 | Bx064_216 | before 2011 | in 2012 | in 2013 | before 2011 | in 2012 | in 2013 | before 2011 | in 2012 | in 2013 | before 2011 | in 2012 | in 2013 | before 2011 | in 2012 | in 2013 | before 2011 | in 2012 | in 2013 | before 2011 | in 2012 | in 2013 | Phenotype ^(c)^ |
| A | A | A | A | A | A | H |  |  |  |  |  |  |  |  |  |  |  |  |  |  |  |  |  |  |  |  |  | PF |
|  |  |  |  |  |  |  | 1 |  |  |  |  |  |  |  |  |  | 1 | 5 |  |  | 2 |  |  |  |  |  |  | SF |
| A | A | A | A | H | H | H |  |  |  |  |  |  |  |  |  |  |  |  |  |  |  |  |  |  |  |  |  | PF |
|  |  |  |  |  |  |  | 1 | 1 |  |  |  |  |  |  |  |  |  | 2 |  |  |  |  |  |  |  |  |  | SF |
| A | A | A | H | H | H | H |  |  |  |  |  |  |  |  |  |  |  |  |  |  |  |  |  |  |  |  |  | PF |
|  |  |  |  |  |  |  |  |  |  |  |  |  |  |  |  |  |  | 1 |  |  |  |  |  |  |  |  |  | SF |
| A | A | H | H | H | H | H | 6 | 3 | 1 |  | 4 |  | 2 |  |  |  | 1 | 1 |  | 1 |  |  |  |  |  |  |  | PF |
|  |  |  |  |  |  |  | 4 |  | 1 |  | 1 |  | 1 |  |  |  |  | 4 |  | 1 | 1 |  |  |  |  |  | 1 | SF |
| A | H | H | H | H | H | H |  | 1 | 1 |  |  |  | 3 |  |  |  |  | 3 |  | 1 | 1 |  |  |  |  |  |  | PF |
|  |  |  |  |  |  |  |  |  |  |  |  |  |  |  |  |  |  |  |  |  |  |  |  |  |  |  |  | SF |
| H | A | A | A | A | A | A |  |  |  |  |  |  |  |  |  |  |  |  |  |  |  |  |  |  |  |  |  | PF |
|  |  |  |  |  |  |  |  |  | 2 |  | 1 |  |  |  |  |  |  | 2 |  |  |  |  |  |  |  |  |  | SF |
| H | H | A | A | A | A | A |  |  |  |  | 1 |  | 1 |  |  |  | 2 |  |  | 9 |  |  |  |  |  |  |  | PF |
|  |  |  |  |  |  |  | 3 |  |  |  | 1 |  | 1 |  |  |  | 2 | 3 |  | 2 |  |  |  | 1 |  |  |  | SF |
| H | H | H | A | A | A | A |  |  | 1 |  |  |  |  |  |  |  |  | 2 |  | 2 |  |  |  |  |  |  |  | PF |
|  |  |  |  |  |  |  |  |  |  |  |  |  |  |  |  |  |  |  |  |  |  |  |  |  |  |  |  | SF |
| H | H | H | H | A | A | A | 2 |  | 1 |  | 2 |  |  |  |  |  | 2 |  |  |  |  |  |  |  |  |  |  | PF |
|  |  |  |  |  |  |  |  |  |  |  |  |  |  |  |  |  |  |  |  |  |  |  |  |  |  |  |  | SF |
| H | H | H | H | H | A | A |  |  |  |  |  |  |  |  |  |  |  | 1 |  |  |  |  |  |  |  |  |  | PF |
|  |  |  |  |  |  |  |  |  |  |  |  |  |  |  |  |  |  |  |  |  |  |  |  |  |  |  |  | SF |
| H | H | H | H | H | H | A |  |  |  |  |  |  |  |  |  |  |  | 3 |  |  |  |  |  |  |  |  |  | PF |
|  |  |  |  |  |  |  |  |  |  |  |  |  |  |  |  |  |  |  |  |  |  |  |  |  |  |  |  | SF |
|  |  |  |  |  |  |  | 29 | | | 10 | | | 8 | | | 35 | | | 20 | | | 1 | | | 1 | | | 104 |

^(a)^ The number of individuals for each site of recombination. H and A, presence and absence of the marker respectively.

^(b) ‘^MdB’ means ‘Mara des Bois’.

^(c)^ Phenotyping for fine mapping was performed before this study or on the seedlings obtained in 2011 and phenotyped in 2012. Seedlings obtained in 2013 have been phenotyped during the summer of 2014.

**Supplemental Table 3.** List of 79 cultivars or selections tested for presence (1) or absence (0) of two SSR markers that flanked the *FaPFRU* locus. Pedigree, year of cultivar release, country of origin, and year of sampling are included.

| Genotype | Pedigree | Year of cultivar relase | Origin | Sampling Year (Douville, FR) ^a^ | Bx083_206 | Flowering Behavior ^b^ | Bx215_131 |
| --- | --- | --- | --- | --- | --- | --- | --- |
| Addie | Pantagruella × MdUS 3816 | 1982 | IT | 2009 | 1 | SF | 1 |
| Agathe | (synonym of Agathe) | 1992 | USA, FL | 2009 | 0 | SF | 0 |
| Alaska Pionner | Senga Sengana × Alaska 292 | 1960 | USA, AK | 2005 | 1 | PF | 1 |
| Albritton | Southland selfed × Massey selfed | 1946 | USA, NC | 2005 | 0 | SF | 0 |
| Appolo | NC 1759 × Nc 1729 | 1970 | USA, NC | 2005 | 0 | SF | 0 |
| Arking | Cardinal × Ark 543 1 | 1981 | USA, AK | 2009 | 0 | SF | 0 |
| Betty | Pajaro × CF206 | 2007 | FR | 2009 | 0 | SF | 0 |
| Blackmore | Missionary × Howard 17 | 1925 | USA, NC | 2005 | 1 | SF | 1 |
| Blanche ananas | - | 1860 | USA | 2009 | 0 | SF | 0 |
| Bogota | Zb.53.116 × Tao | 1977 | NL | 2000 | 0 | SF | 0 |
| Bounty | Jerseybelle × S. Sengana | 1972 | USA, CA | 2000 | 0 | SF | 0 |
| Brighton | Tufts × CAL 65.65-601 | 1979 | USA, CA | 2005 | 1 | PF | 1 |
| Camarosa | Douglas × Cal 85.218-605 | 1992 | USA, CA | 2005 | 0 | SF | 0 |
| Candiss | CF1713 X Allstar | 2008 | FR | 2009 | 1 | SF | 1 |
| Candonga | Sel. 9238 × Sel. 86032 | 2003 | SP | 2005 | 0 | SF | 0 |
| Capitola | CN 25 [ = Cal 75.121-101]×Parker | 1992 | USA, CA | 1999 | 1 | PF | 1 |
| Catskill | Marshall × Howard 17 | 1934 | USA, NY | 2000 | 1 | SF | 0 |
| CF1116 | Pajaro × (Earlyglow × Chandler) | 1998 | FR | 1999 | 0 | SF | 0 |
| CF129 | Earlyglow × Chandler | - | FR | 1999 | 1 | SF | 1 |
| CF1778 | - | - | FR | 2005 | 0 | SF | 0 |
| CF3058 | - | - | FR | 2005 | 0 | SF | 0 |
| CF3453 | - | - | FR | 2009 | 0 | SF | 0 |
| Chandler | Douglas × Klon C 55 or Douglas × CAL 72.361-105 | 1980 | USA, CA | 1999 | 0 | SF | 0 |
| Charlotte | Mara des Bois × Cal 19 | 2004 | FR | 2009 | 1 | PF | 1 |
| Ciflorette | Mamie × Earlyglow | 1998 | FR | 2009 | 1 | SF | 1 |
| Cigaline | Gariguette × Earlyglow | 1998 | FR | 2009 | 0 | SF | 0 |
| Cijosée | Mara des Bois × Cal. 18 | 1997 | FR | 2009 | 0 | PF | 1 |
| Cilady | Scoot × Chandler | 1996 | FR | 2005 | 0 | SF | 0 |
| Cirafine | Mara des Bois × Cal. 18 | 1998 | FR | 2009 | 1 | PF | 1 |
| Cirano | Mara des Bois × Muir | 1997 | FR | 2005 | 1 | PF | 1 |
| Ciréine | Scott × Chandler | 1996 | FR | 2005 | 1 | SF | 1 |
| Clery | Sweet Charlie × Marmolada® Onebor | 2002 | IT | 2005 | 0 | SF | 0 |
| Dalton | - | - | - | 2005 | 0 | SF | 0 |
| Darselect | Elsanta × Parker | 1996 | FR | 2009 | 0 | SF | 0 |
| Darsidor | Aiko × Selecta | 1992 | FR | 2005 | 0 | SF | 0 |
| Diamante | CAL 87.112-6 × CAL 88.270-1 | 1995 | USA, CA | 2005 | 1 | PF | 1 |
| Divine | - | 2004 | FR | 2005 | 0 | SF | 0 |
| Docteur Morère | Duc Malakoff × Palmyre Berger | 1871 | FR | 2005 | 0 | SF | 0 |
| Donner | CAL. 145.52 × CAL 222 | 1945 | USA, CA | 2005 | 0 | SF | 0 |
| Dover | Floridabelle × FLA 71 189 | 1980 | USA, FL | 1999 | 0 | SF | 0 |
| Earlyglow | MdUS 2359 [Fairland×Midland] × MdUS 2713 [Redglow×Surecrop] | 1975 | USA, MD | 2009 | 1 | SF | 1 |
| Elsanta | Gorella × Holiday | 1981 | NL | 1999 | 1 | SF | 1 |
| Elsinore | (Elsanta x Muir) × Sweet Charlie | - | IT | 2005 | 1 | PF | 1 |
| Emily | Honeoye × Gea | 1995 | GB | 2009 | 0 | SF | 1 |
| Favette | (Surprise des Halles × Regina) × (Pocahontas × Aliso) | 1976 | FR | 2008 | 0 | SF | 0 |
| Frel | *P. palustris* × *F. x ananassa* | 1990 | UK | 2008 | 1 | PF | 1 |
| Gariguette | (Pocahontas x Regina) × (Belrubi x Marieva) | 1972 | FR | 2009 | 0 | SF | 0 |
| Heidi | Driscoll E3 × Driscoll NM55.5 | 1965 | USA, CA | 2005 | 1 | SF | 0 |
| Hokowase | - | - | JPN | 2005 | 1 | PF | 0 |
| Honeoye | Tamella × Induka | 1985 | USA, NY | 2005 | 0 | SF | 0 |
| Louis Gauthier | Belle de Meaux × Marguerite Lebreton | 1896 | FR | 2005 | 1 | SF | 1 |
| Madeleine | - | 1998 | IT | 2005 | 0 | SF | 0 |
| Mamie | Harvester × Gariguette | 1988 | FR | 2009 | 0 | SF | 0 |
| Mara des Bois | (Hummi Gento x Ostara) × (Red Gauntlet x Korona) | 1992 | FR | 2009 | 1 | PF | 1 |
| Mars | IA 81-6733 × IA 22-6014 | - | USA, IA | 2005 | 0 | SF | 0 |
| Matis | - | 2003 | FR | 2005 | 0 | SF | 0 |
| Naiad | Oso Grande × Eris®Civero | 2000 | IT | 2005 | 0 | SF | 0 |
| Nova Gento | - | - | - | 2009 | 1 | PF | 1 |
| Nyoho | Kei 210 × Reïko | 1984 | JPN | 2009 | 1 | SF | 0 |
| Oso Grande | Parker × CAL 77.3-603 | 1982 | USA, CA | 2000 | 0 | SF | 0 |
| Ostara | Redgauntlet × Macherauchs Dauerernte | 1969 | NL | 2005 | 1 | PF | 0 |
| Pajaro | Sequoia × C 63 7 101 | 1979 | USA, CA | 1999 | 0 | SF | 0 |
| Parker | Douglas × (Tufts x 63.7.101) | 1983 | USA, CA | 1999 | 0 | SF | 0 |
| Redgauntlet | NewJersey1051 × Auchincruive Climax | 1957 | GB | 2005 | 0 | SF | 0 |
| Revada | Auschincruive Climax × Ada Herzberg | 1956 | NL | 2000 | 1 | PF | 1 |
| Rosanne | self-pollination of NC 3140 | 1969 | USA, NC | 2005 | 1 | SF | 0 |
| Saint Joseph | - | - | FR | 2009 | 0 | SF | 0 |
| Selva | CAL 70.3-117 (sister of Brighton) × (Tufts x 63.7-101 (parent of Pajaro) | 1977 | USA, CA | 2000 | 1 | PF | 1 |
| Sequoïa | CAL 52.16-15 × CAL 51s1-1 | 1968 | USA, CA | 1999 | 0 | SF | 0 |
| Soquel | Cruz × Aïko | 1983 | USA | 2005 | 0 | SF | 0 |
| Stoplight | (Florida Ninety x Cyclone) x 6-5908 | 1964 | USA, IA | 2005 | 0 | SF | 0 |
| Sweet charlie | FL 80-456 × Pajaro | 1992 | USA, FL | 2008 | 0 | SF | 0 |
| Späete Leopolod | Comet × Sämling | 1920 | GE | 2008 | 0 | SF | 0 |
| Tribute | (MdUS 3082 × CAL 65.65-601) × (MdUS2713 × MdUS 3364) | 1975 | USA, MD | 2009 | 1 | PF | 1 |
| Trumpeter | Burgundy × SYS 3672179 (seedling of Howard 17) | 1946 | USA, MN | 2005 | 1 | SF | 0 |
| US159 | - | 1992 | USA, MD | 1999 | 0 | SF | 0 |
| US292 | - | 1992 | USA, MD | 1999 | 0 | SF | 0 |
| US70 | - | 1992 | USA, MD | 1999 | 0 | SF | 0 |
| Valeta | Sivetta × Holiday | 1983 | NL | 2000 | 0 | SF | 0 |

^a^, Year of DNA extraction. Leaves of cultivars and selections were all sampled in Douville, France.

^b^, Seasonal Flowering (SF) or Perpetual Flowering (PF): data from observations of GenBerry project (unpublished data), National Germplasm Repository of Corvallis (<http://www.ars.usda.gov/main/site_main.htm?modecode=20-72-15-00>) or Darrow (1966).

**Supplemental Table 4.** List of the SSR primer pairs used in this study.

| ^SSR name^ | ^Forward primer^ | ^Reverse primer^ | ^SSR motif^ | ^Expected size (bp)^ | ^Position on scf0513158 (v1.0)^ |
| --- | --- | --- | --- | --- | --- |
| ^Bx089^ | ^CACCAAAGATGACTGCTGGA^ | ^TAAACAGCCCCTGAATCCAA^ | ^(AATCC)4^ | ^225^ | ^2275082 - 2275307^ |
| ^Bx083^ | ^ACGTGCCTTAGCGGATCATA^ | ^CCTAGGTCGGGATCTCAGAA^ | ^(AG)18^ | ^217^ | ^2347057 - 2347274^ |
| ^Bx215^ | ^CAATTTCCCGCCAAAAGTAA^ | ^GTTGGAGCTTCGAGCAAGTT^ | ^(AG)20^ | ^151^ | ^3454762 - 3454913^ |
| ^Bx052^ | ^ACCAGCCTGCTGCTGTAGTT^ | ^ACCACCTCCACATTCCACAT^ | ^(AAG)5^ | ^236^ | ^3478525 - 3478761^ |
| ^Bx059^ | ^GACGTTGACCATGACAGAGC^ | ^CCCAAAGAAAGCCCAACATA^ | ^(ATC)9^ | ^258^ | ^3695856 - 3696114^ |
| ^Bx056^ | ^GGTTACTGGCTCTGCTTGGA^ | ^CACAATTGAGATCGAGCAACA^ | ^(AAG)5^ | ^209^ | ^3593523 - 3593732^ |
| ^Bx064^ | ^GGGGAGGTGAAACTGTGAAA^ | ^TGCAATCTTTGGGAGAGAGAA^ | ^(AAG)7^ | ^222^ | ^3876118 - 3876340^ |
| ^Bx250^ | ^GGCATTTCCGCAGATAAAAA^ | ^AACCTCCTCGTGTTTGATGC^ | ^(AAG)6^ | ^230^ | ^4218509 - 4218739^ |
